# Smart Glasses for Older Adults With Cognitive Impairment: A Scoping Review

**DOI:** 10.1016/j.jamda.2025.105831

**Published:** 2025-09-11

**Authors:** Brittany F. Burch, Jinwei Gu, Gail Betz, Chien-Ming Huang, Rachel McPherson, Alice S. Ryan, Barbara Resnick

**Affiliations:** a School of Nursing, University of Maryland, Baltimore, Baltimore, MD, USA; b Health Sciences and Human Services Library, University of Maryland, Baltimore, Baltimore, MD, USA; c Whiting School of Engineering, Johns Hopkins University, Baltimore, MD, USA; d School of Medicine, University of Maryland, Baltimore, Baltimore, MD, USA

**Keywords:** Smart glasses, older adults, cognitive impairment, technology, dementia

## Abstract

**Objectives:**

This study aimed to identify and synthesize peer-reviewed literature on smart glasses for older adults with cognitive impairment. The review focused on (1) the technology and equipment used, (2) the specific tasks smart glasses are designed to support, (3) feasibility outcomes, when measured, and (4) the quality of the reviewed articles.

**Design:**

Scoping review.

**Setting and Participants:**

Older adults with cognitive impairment.

**Methods:**

A research librarian conducted a comprehensive search across 8 databases to identify peer-reviewed studies that investigated the use of smart glasses for older adults with cognitive impairment. Two authors independently used Covidence to review articles for extraction and organization.

**Results:**

Our search yielded 414 articles and 13 were included for data extraction and appraisal. All included studies were rated as low quality. Seven studies focused on commercially available smart glasses and 6 focused on early-stage prototypes of smart glasses. Only 5 studies involved hands-on testing with older adults, and just one study exclusively involved participants with cognitive impairment. The main purpose of the smart glasses included assisting older adults with daily tasks, recognizing faces, aiding in navigation, and helping care partners locate or monitor individuals with cognitive impairment.

**Conclusions and Implications:**

More rigorous studies are needed to test smart glasses among older adults with cognitive impairment and the literature does not reflect the recent advancements in smart glasses technology. Future smart glasses research should include more interdisciplinary teams, utilize user-centered design frameworks, and obtain long-term feasibility and effectiveness data among larger samples of older adults with cognitive impairment.

One in 5 older adults is affected by cognitive impairment,^1^ a condition characterized by difficulties in understanding, remembering, and making decisions. Cognitive impairment varies in severity, ranging from mild cognitive impairment, which can be potentially reversible, to severe dementia. Most cases of cognitive impairment are progressive, and many are associated with increased depression and mortality.^2,3^ Despite its high prevalence and burden, no medical cure exists, and current treatment options demonstrate limited effectiveness.^4,5^ Therefore, interventions that can help older adults *live with* their cognitive impairment are critical.

## Technology-Based Solutions for Older Adults With Cognitive Impairment

Technological advancements have greatly improved the lives of older adults with cognitive impairment. For example, computerized cognitive training has been associated with improvements in short-term memory, executive function, and processing speed in both healthy and cognitively impaired older adults.^6^ Fully-immersive virtual reality interventions, such as reminiscence therapy, have been linked to increased engagement and potentially improved mental health.^7,8^ In addition, sensor-based monitoring of daily tasks has been used to aid in diagnosing cognitive impairment and enabling care partners to remotely monitor their care recipients (individuals with cognitive impairment).^9^ In the past decade, and most pertinent to this review, researchers have begun to explore the role of smart glasses in assisting older adults with cognitive impairment.

## Smart Glasses for Older Adults With Cognitive Impairment

For the purposes of this paper, smart glasses are defined as “an eye-worn device that provides function(s) similar to a computer to assist the wearer.”^10^ Similar to smartwatches or smart rings, smart glasses integrate a commonly worn device (ie, glasses) with features that absorb and relay user and/or environmental information. Smart glasses vary greatly in complexity. Basic smart glasses function mainly as phone accessories, assisting with calls or access to a virtual assistant (eg, Amazon Echo Frames). More advanced smart glasses utilize sophisticated artificial intelligence (AI) features that would typically require human intelligence such as helping the user identify or interpret their surroundings (eg, Ray Ban Meta Glasses). Recently, some smart glasses have been developed with augmented reality capabilities, allowing projections superimposed on the glasses lens. These smart glasses with augmented reality capabilities offer features such as navigation displays, speech to text language translation, and virtual screens for entertainment (eg, Vuzix Z100 Glasses). The distinction between smart glasses with augmented reality capabilities and augmented reality headsets is primarily based on *subjective* factors such as portability and minimalism. The Microsoft HoloLens, classified in literature as both smart glasses and an augmented reality headset, exemplifies this ambiguity.

## Previous Literature and Research Gap

Although previous reviews related to smart glasses have been conducted, there remains a gap in synthesizing recent research, particularly regarding smart glasses for older adults with cognitive impairment. Kim and Choi^11^ reviewed all smart glasses literature between 2014 and 2020, encompassing a broad range of populations and fields. This review found that the most popular commercial smart glasses used in research were Android and Google-based smart glasses and that smart glasses were most commonly used in health research for people with disabilities. However, their review did not focus on smart glasses for cognitively impaired individuals. Blattgerste et al^12^ reviewed augmented reality technology for cognitively impaired individuals, focusing only on action assistance and learning. This review found that individuals with cognitive impairment generally accepted augmented reality technologies, but emphasized the need for studies with larger, more diverse samples and long-term follow-up and data collection. Blattgerste et al^12^ did not *specifically* focus on glasses (eye-based) technology. In addition, this literature review was performed in 2018 and thus predates the recent surge in smart glasses technology. Although most studies in their review^12^ used handheld devices and projectors, the authors explicitly predicted that smart glasses would be the future research focus of technology interventions for individuals with cognitive impairment.

## Significance and Aims

Smart glasses present a promising technological solution to help older adults with cognitive impairment manage their memory-related challenges. Unlike other technologic interventions, such as robots or tablet-based cognitive training, many older adults are already accustomed to wearing and carrying glasses in their daily lives. In addition, smart glasses offer many hands-free features that may be more intuitive for older adults experiencing memory difficulties. Given the high burden of cognitive impairment among older adults and the recent advancement of smart glasses research and development, there is a pressing need for a comprehensive review of the literature on this topic. This study aimed to fill this research gap by identifying and synthesizing the peer-reviewed literature on smart glasses for older adults with cognitive impairment, with a content focus on (1) the technology and equipment used, (2) the specific tasks that smart glasses are designed to support, (3) feasibility outcomes, when measured, and (4) the quality of the reviewed articles. By addressing these areas, this review provides valuable guidance for smart glasses developers, researchers, and clinicians.

## Methods

This scoping review follows the methodology as described by the PRISMA-ScR checklist and is registered with the Open Science Framework (DOI 10.17605/OSF.IO/X64FS), a free and open-source project management tool.

### Inclusion and Exclusion Criteria

The inclusion criteria were studies that (1) investigated the use of smart glasses, which were defined as “an eye-worn device that provides function(s) similar to a computer to assist the wearer”; (2) stated a target demographic for the glasses as older adults with cognitive impairment, regardless of whether they tested the glasses among this population; (3) were a peer-reviewed article, book, or conference paper; and (4) were written or translated into the English language. We required no sample age range for article inclusion, provided the article stated that the population focus was older adults. We excluded studies that (1) were case reports or (2) utilized headsets or goggles, rather than glasses. As smart glasses are a novel technology, we set no restrictions on publication date.

### Literature Search and Organization

On April 10, 2024, we searched the following databases: Scopus, Ovid Medline, EBSCOhost (PsychINFO, Ageline, Abstracts in Social Gerontology), ACM Digital Library, Embase, and Cochrane Library. A research librarian (G.B.) developed the search strategy, which can be found in the Supplementary Material. The search strategy included relevant terms for smart glasses, cognitive impairment, and older adults. A second research librarian reviewed the search strategy for accuracy.

**Fig. 1.**
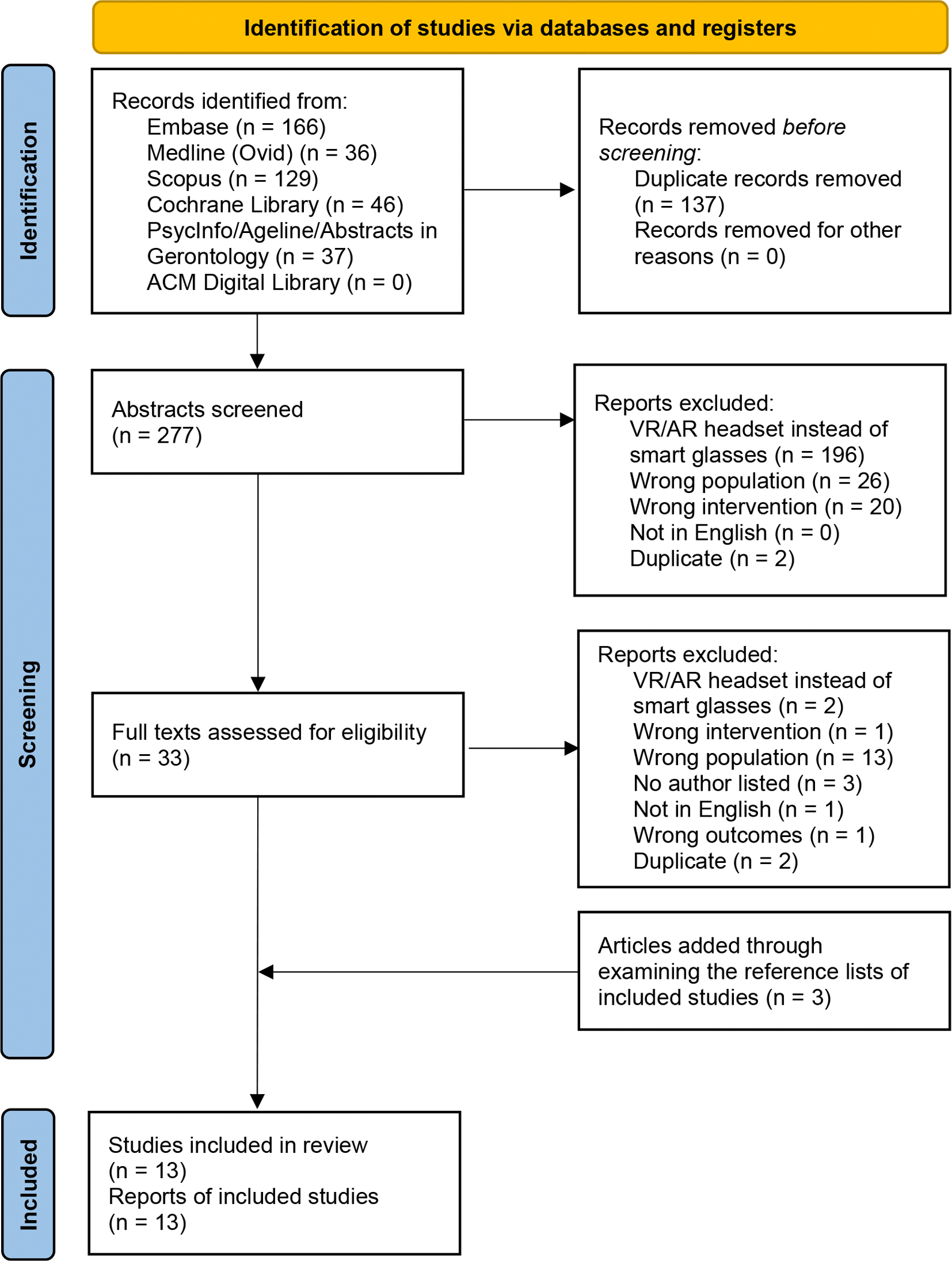
The PRISMA Flow Diagram describes the article screening process. The search generated 414 articles, 137 of which were duplicates and subsequently removed. We removed 244 articles during the title and abstract screening and we removed an additional 23 articles during the full-text screening, resulting in 10 articles remaining. On reviewing the reference lists of the 10 remaining articles, 3 additional articles were included, resulting in a total of 13 articles for this review. VR/AR, virtual reality/augmented reality.

Two authors (B.F.B. and J.G.) independently used Covidence, an article organization software, to review articles and determine exclusion.^13^ This review process included removal of duplicates, abstract screening, and full-text screening (Figure 1, PRISMA Diagram). After articles were selected for review, the same 2 authors (B.F.B. and J.G.) independently extracted information from the articles using a structured synthesis table and compared results for accuracy and consistency. The extracted information included the following: first author; year of publication; dissemination type; sample size; sample description; type of cognitive screening tool, as applicable; comparison group, as applicable; glasses type; main purpose of glasses; tasks performed by participants using the glasses, as applicable; outcomes related to the glasses testing, as applicable; and the quality appraisal of each article. During the extraction process, the authors made refinements to the synthesis table. Specifically, they combined 2 columns, main purpose of glasses and tasks performed by the participants, due to content overlap. In addition, they removed 2 columns, type of cognitive screen and comparison group, as most included studies emphasized the development and feasibility of smart glasses, rather than comparative testing among samples with cognitive impairment. A simplified version of this data extraction table is presented in the results section.

### Article Appraisal

B.F.B. evaluated the included studies for methodological quality using the Effective Public Health Practice Project Quality Assessment Tool. The Effective Public Health Practice Project Quality Assessment Tool is a validated appraisal tool that enables researchers to assess the quality of studies based on 6 domains: selection bias, study design, treatment of confounders, blinding, data collection methods, and drop out.^14,15^ This is a strong tool for appraising studies with varying designs within an emerging field of research.

## Results

### Samples and Screening

The search generated 414 articles, 137 of which were duplicates and subsequently removed. We removed 244 articles during the title and abstract screening and we removed an additional 23 articles during the full-text screening, resulting in 10 articles remaining. The most common reason for exclusion was a focus on virtual reality or augmented reality headsets, rather than smart glasses. On reviewing the reference lists of the 10 remaining articles, 3 additional articles were included, resulting in a total of 13 articles for this review (see Table 1). Of note, the article screening process yielded 5 article inclusion discrepancies, which the 2 screeners (B.F.B. and J.G.) resolved through discussion. See Figure 1 for the PRISMA Diagram detailing the article screening process.

### Technology Used

Among the 13 included articles, 3 articles focused on HoloLens glasses,^16–18^ one on Google Glass,^19^ one on Optinvent ORA-2 glasses,^20^ and one on SensoMotoric Instruments eye tracking glasses.^21^ The remaining articles either did not specify the commercial glasses used^22^ or proposed/developed prototype smart glasses for the purpose of the study.^23–28^ Prototypes were generally rudimentary and bulky such as regular glasses with components added to the side arm of the glasses frame, such as camera or video features,^24,27,28^ microphones,^25^ accelerometers,^23,24,28^ a GPS module,^27^ eye tracking or sensors,^25^ and indicator lights.^25,26^

### Purpose of the Smart Glasses

The primary purpose of the smart glasses proposed or tested varied across studies. Seven studies explored smart glasses use for assisting with daily tasks, such as calling loved ones,^19,25^ detecting misplaced objects,^24^ identifying dangers,^22,28^ and aiding in manual activities such as cooking.^17,21^ Two studies explored smart glasses for the purpose of face recognition^24,27^ and 5 studies explored smart glasses for the purpose of assisting older adult users with navigation assistance through projected lens indicators^16,19,20^ or frame-based lights.^25,26^ In addition, 4 studies focused on smart glasses as tools for care partners, providing location tracking^24,27^ and behavioral insights.^23,28^

### Testing and Outcomes

Among the 13 included articles, 7 studies described smart glasses development^23–27^ or described the theoretical/proposed use of smart glasses without actual testing.^18,22^ Only 6 studies^16,17,19–21,28^ involved smart glasses testing–collecting data from individuals wearing them. Although all articles included in this review described “older adults with cognitive impairment” as a potential target population for the smart glasses per the inclusion criteria, only 1 of the 6 studies tested the glasses exclusively among older adults who had cognitive impairment.^17^ However, 5 studies^17,19–21,28^ tested the glasses among older adults. These 5 studies are summarized in the following and mainly report usability and acceptability of the smart glasses.

First, Haesner et al^19^ evaluated Google Glass with 30 older adults performing standardized tasks such as sending a picture, taking a phone call, or obtaining navigational directions. Although participants made errors and required assistance, the participants reported marginal to good usability (User Experience Questionnaire = positive rating for all domains; System Usability Scale = 64.2, SD = 14.1). Their primary concern with the smart glasses was an increased fear of falling. Of note, several older adult participants were found to have mild cognitive impairment; this subgroup needed significantly more time to conduct the tasks, as well as more assistance. Second, Essig et al^21^ tested SensoMotoric Instruments glasses among “handicapped and elderly” participants (sample size not stated) while assembling a birdhouse or using a modern coffee machine. Participants appreciated the legibility and usefulness of the visual hints superimposed on the glasses lens but criticized the weight of the glasses. Third, Wolf et al^17^ examined HoloLens smart glasses in a cooking task among 6 older adults with mild cognitive impairment. Compared with paper recipe—assisted tasks, the glasses-assisted tasks took longer to complete, but the participants required less external help. Participants reported positive technology affinity scores (Affinity for Technology-Electronic Gadgets [TAEG] positivity score = 3.93, SD = 0.68) but noted a “somewhat high” cognitive workload (NASA task load index [NASA-TLX] workload = 40.83, SD = 12.8). Fourth, Zhan et al^28^ tested their recently developed glasses prototype for the purpose of recognizing the daily activities of older adults, finding that the glasses technology could recognize activities with up to 90% accuracy for older adults, and 77% for those with disabilities (eg, musculoskeletal, neurological, weakness). Participants reported positive qualitative feedback related to the glasses comfort but clarified that the comfort is limited to short periods of time due to weight. Last, Montuwy et al^20^ compared navigation performance among 18 older adults using 4 different aids: usual navigation aid (eg, paper map), Optinvent ORA-2 augmented reality glasses, headphones, and smartwatches. Although navigation time was significantly shorter with the augmented reality glasses compared with the usual aid, participants had significantly lower success at intersections when using the glasses. Participants also expressed qualitative concerns about distractions and the conspicuous nature of the glasses and stated that they were more likely to wear the comparison devices (ie, headphones or smartwatch) to navigate the city.

## Article Appraisal

We rated the 7 studies that only described the development or theoretical use of a pair of smart glasses without any prototype testing as low-quality studies.^18,22–27^ According to the Effective Public Health Practice Project Quality Assessment Tool, all remaining 6 studies^16,17,19–21,28^ that tested the glasses among participants also received a quality rating of low. These studies were generally rated poor in selection bias because of low sample size (n = 4–30) and poor in study design because of lack of a comparison group.^16,19,21,28^

## Discussion

This review included 13 articles that investigated smart glasses for older adults with cognitive impairment. Although 7 studies focused on existing smart glasses that were commercially available, the remaining 6 focused on early-stage prototypes of glasses. Across all 13 studies, the main purpose of the smart glasses included assisting older adults with daily tasks, recognizing faces, aiding in navigation, and helping care partners locate or monitor individuals with cognitive impairment. Most studies described the development or theoretical/proposed use of the pair of smart glasses without actual testing. The 5 studies that tested the glasses among older adults primarily reported usability and acceptability outcomes. Taken together, these 5 studies indicate that although older adults may make errors and, in some cases, take longer to perform tasks while using smart glasses, they generally report marginal to good usability and acceptability. However, older adults suggest that improvements in the quality of smart glasses are needed before long-term integration into their daily lives. Specifically, improvements should focus on minimizing weight, ensuring glasses use is intuitive, and preventing visual obstruction (as applicable).

Among studies exploring the development of smart glasses for older adults with cognitive impairment, we have identified research gaps and areas of future study. First, when describing the development of smart glasses, authors should provide a detailed step-by-step account of their design and functions to contribute meaningfully to research progression. As an example, Saleh and colleagues^27^ thoroughly and transparently describe the design, components, and development of the system hardware such as the camera, processor, ultrasonic sensor, and GPS module, as well as the overall circuit connection. Second, future development research should explore the integration of smart glasses with other assistive technologies or household appliances. For instance, smart glasses could be linked to smart doors or ovens to detect when an older adult with cognitive impairment forgets to lock a door or turn off an oven. In the context of post-acute and long-term care settings, smart glasses could assist care staff in monitoring residents’ locations and behaviors while preserving resident independence. Last, future development research should include more interdisciplinary teams and user-centered design frameworks, similar to that of Essig et al.^21^ Collaboration among clinicians, computer scientists, and older adults experiencing memory issues could help ensure that smart glasses align with the needs and values of the intended users.

Commercial smart glasses have evolved significantly in the past decade, yet are not represented in the current literature. Modern smart glasses such as Ray Ban Meta Glasses, Amazon Echo Frames and Vuzix Z100 Glasses feature minimalistic designs, improved display and audio technology, and/or AI-powered capabilities. As smart glasses technology continues to advance and augmented reality headsets/goggles become more compact, these technologies are expected to merge. Unfortunately, the current literature does not reflect recent advancements in smart glasses technology. Most studies included in this review were published between 2016 and 2019 and, therefore, focused on earlier generations of commercial smart glasses such as the Google Glass and HoloLens. This review underscores the urgent need for research on how modern iterations of smart glasses could be leveraged or adapted to assist older adults living with cognitive impairment.

Although the studies that assessed usability and acceptability outcomes generally reported marginal to good usability and acceptability, these findings should be interpreted with caution. Notably, the only study that compared smart glasses with other technological intervention(s), Montuwy et al,^20^ reported the most negative participant feedback regarding the smart glasses. Participants verbalized a preference for the comparison devices (ie, headphones or smartwatch) in navigating a city, rather than the smart glasses.^20^ This finding brings into question whether the modestly positive usability outcomes reported in the other studies might have been attenuated had comparison groups been included. Alternatively, the negative results reported by Montuwy et al^20^ may have stemmed from the nature of the studied task. As smart glasses are an eye-worn device, it is unsurprising that a display on the glasses lens was perceived as distracting while navigating an urban environment. Ultimately, the findings from this study highlight a need for careful interpretation of smart glasses literature, as usability can vary significantly based on study design and technological differences between products.

This review indicates that more rigorous studies are needed to test smart glasses specifically among older adults with cognitive impairment. Consistent with the scoping review methodology, our approach was designed to broadly capture the full scope of research activity in this emerging field, including developmental studies. Scoping reviews are particularly suited to research topics that are limited or still evolving and can assist with informing future work within the emerging field. Although developmental or theoretically focused studies provide important insights, it is essential to also test and obtain feedback from those for whom the technology is designed. Going forward, researchers should prioritize implementation-focused studies and studies that examine the long-term feasibility of smart glasses among larger samples of older adults with varying levels of cognitive impairment. Such studies could better assess smart glasses use amidst the complexities of daily life use, including long-term wear comfort and adherence over time. In addition, integrating prescription-based lenses, when applicable, would enhance the day-to-day relevance of these studies. In addition to feasibility and acceptability outcomes, researchers should also move toward examining long-term effectiveness. For example, researchers could investigate the effectiveness of smart glasses at supporting aging in place through improved medication adherence (eg, glasses offering medication reminders), improved social support (eg, glasses used for calling loved ones), and/or decreased hospitalizations/long-term care placements.

Although professional workers (eg, maintenance workers, health care workers) and the general population have been increasingly adopting smart glasses for training, work assistance, disability support, or everyday life, this adoption is more prevalent among younger individuals.^11,29–31^ To support the acceptance and usability among older adults, including those with cognitive impairment, developers must consider the features that older adults find beneficial, while also addressing risk mitigation strategies such as approaches to minimize falls. Sedighi et al^32,33^ found that smart glasses equipped with augmented reality technology (eg, lens display) affected users’ perceptions of stability. Given this information, older adults with mobility impairment might be better suited for smart glasses without augmented reality technology to prevent visual obstructions or distractions. If smart glasses are indeed developed with augmented reality technology, the reviewed studies by Montuwy et al^20^ and Haesner et al^19^ suggest that design efforts should prioritize sharp images and reduced visual obstruction if use is intended while standing.

Additional ethical and practical considerations for developers and researchers of smart glasses for older adults with cognitive impairment might include reducing cognitive burden and ensuring privacy and informed consent. As only one study from this review tested the glasses specifically among older adults who had cognitive impairment,^17^ we cannot draw conclusions about whether smart glasses contribute to confusion in this population. In future work, smart glasses developers and researchers should assess whether added safety benefits outweigh potential new risks introduced from their use. For example, the usefulness of smart glasses to recognize environmental hazards could be undermined if their usage incites confusion or behavioral symptoms in the wearer. In the context of privacy, researchers must be fully transparent regarding what data will be collected and used for research purposes, and how these data will be thoughtfully protected. In addition, researchers should educate the older adults and/or their caregivers on responsible use such as protecting the privacy of bystanders and how to manage the glasses’ data collection settings, as applicable. Last, researchers should obtain consent from older adults with cognitive impairment who can provide it. Older adults with mild cognitive impairment often have the capacity to understand, evaluate, and make decisions about their technology use and involvement in research. For those who lack capacity, researchers should obtain assent from the older participant and consent from care partners, ensuring ongoing monitoring of participant willingness to continue glasses use throughout the course of the study.

## Strengths and Limitations

This review has several limitations and strengths. First, the number of studies testing smart glasses among older adults, especially those with cognitive impairment, was small. Second, all of the included studies were identified as low methodological quality studies. These limitations restrict the ability to draw broad conclusions or generalize findings to the larger population. In addition, review exclusion of non-English studies may have led to the omission of relevant articles. A major strength of this review is its systematic and transparent approach to identifying literature on a novel topic. Furthermore, by including a broad range of studies, including those focused on smart glasses development or theoretical/proposed use, this review provides a comprehensive understanding of the current state of research and highlights key gaps.

## Conclusion and Implications

This review included 13 articles that investigated the use of smart glasses for older adults with cognitive impairment. Although 7 studies focused on existing smart glasses that were commercially available, the remaining 6 focused on early-stage prototypes of glasses. The major conclusion from this review and article appraisal is that more rigorous studies are needed to test smart glasses among older adults with cognitive impairment and the literature does not reflect the recent advancements in smart glasses technology. Future smart glasses research should include more interdisciplinary teams, use user-centered design frameworks, and obtain long-term feasibility and effectiveness data among larger samples of older adults with cognitive impairment.

## Supplementary Material

Supp material

Supplementary Data

Supplementary data related to this article can be found online at https://doi.org/10.1016/j.jamda.2025.105831.

## Figures and Tables

**Table 1:** Synthesis of Studies

Author (Year)	Dissemination Type	Glasses Type	Tested Among Older Adults?	Sample Size (if Applicable)	Main Purpose of Glasses?	Participant Feasibility Outcomes, if Reported

Chen (2018)	Conference paper	Regular eyeglasses with equipment added to frame		N/A	Recognize daily movements (locations and actions) and notify care partner when behavior abnormal	
Essig (2016)	Conference paper	SensoMotoric Instruments eye tracking glasses	X	Handicapped and older individuals, sample size not stated	Provide helpful information projected on the lens to suggest the next appropriate action step for a task (eg, building a birdhouse)	Qualitative results provided only. Participants reported not being substantially physically restricted by the glasses and appreciated the legibility and usefulness of the visual hints superimposed on the glasses lens. Although the participants could imagine wearing the glasses for longer durations, they criticized the glasses weight.
Firouzian (2017)	Conference paper	Regular eyeglasses with equipment added to frame		N/A	Provide speech and visual interface to help complete simple tasks, such as navigation, making calls, or managing appointments	
Gacem (2019)	Conference paper	Regular eyeglasses with equipment added to frame		N/A	Detect the location of misplaced objects, identify and display the names of friends, and detect if patients lost their way and guide them home, while notifying the care partner of the location	
Haesner (2018)	Journal article	Google Glass	X	30 older adults with no or mild cognitive impairment	To perform continuous hands-free tasks, such as taking a picture, getting directions, making calls	Average of 1.5 (SD 1.7) to 3.0 (SD 1.8) errors per task; seniors with cognitive impairment needed more time (b = −0.45, t(28) = −2.63, *P* < .05) and assistance (b = −0.42, t(28) = −2.44, *P* < .05) for the 4 tasks; System Usability Scale 64.2 (SD = 14.1) or “marginal usability”; User Experience Questionnaire revealed positive (above neutral) rating for all domains including dependability, stimulation, perspicuity, attractiveness; 2 participants perceived the display distracting and “many” participants demonstrated difficulty seeing the edges of the display.
Kimura (2017)	Conference paper	HoloLens		4 drivers, unclear if older adults	Assist with controlling and maneuvering welfare vehicles through obstacles such as narrow corridors	
Lääkkö (2014)	Conference paper	Regular eyeglasses with equipment added to frame		N/A	Improve focus and assist in daily activities such as navigation using light indicator cues	
Montuwy (2019)	Journal article	Optinvent ORA-2 glasses	X	18 older adults	Assist with navigation through arrows projected on the lens	Navigation time was significantly longer with the usual aid compared with the augmented reality glasses (M = +27.1%, SD = 16.8, *P* < .005). Success at intersections was significantly lower with the augmented reality glasses (M = 82.5%, SD = 18) compared with the usual aid (*P* < .05). Participants expressed higher attentional workload and lower trust with the augmented reality glasses compared with the other devices. Participants also expressed qualitative concerns about distractions, the conspicuous nature of the glasses, and difficulties perceiving information with the augmented reality glasses (brief, faded).
Rossi (2020)	Conference paper	Not specified		N/A	Provide visual and auditory information for the wearer so that they can independently perform activities of daily living (eg, identifying dangerous situations)	
Saleh (2022)	Conference paper	Virtual reality glasses frame only, with equipment added to frame		N/A	Face recognition, GPS for tracking	
Wolf (2019)	Conference paper	HoloLens	X	6 older adults with mild cognitive impairment	Provide visual and audio prompts to assist with day-to-day manual tasks such as cooking	While completing the HoloLens assisted tasks, participants took 36 min (SD = 9.43) and required help 4 times (SD = 3.2). While completing the paper recipe-assisted tasks, participants took 28 min (SD = 15.45) and required help 6 times (SD = 7.6). The TAEG questionnaire suggests a high positivity score (M = 3.93, SD = 0.68) and low negativity score (M = 2.27, SD = 1.04) toward new technology, but a high NASA-TLX workload (M = 40.83, SD = 12.8).
Yaddaden (2022)	Journal article	HoloLens		29 occupational therapists across 5 focus groups	N/A; Exploratory study to obtain occupational therapist feedback on how smart glasses could help promote older adults’ independence and safety at home	
Zhan (2015)	Journal article	Safety glasses with equipment added to frame	X	5 older adults and 30 individuals of varying ages (34–89 years) with disability	Monitor activities of daily living for the purpose of (1) allowing care partners to track care recipients or (2) warning wearer of hazardous situations	The glasses could recognize activities with up to 90% accuracy for older adults, and 77% for those with disabilities (eg, musculoskeletal, neurological, weakness). Participants reported positive qualitative feedback related to the comfort of the glasses but clarified that the comfort is limited to short periods of time due to weight.
