# Supplementary material for: Smart Glasses for Older Adults With Cognitive Impairment: A Scoping Review
Source: J Am Med Dir Assoc. Author manuscript; Available in PMC 2025 Nov 20. (PMC12633631; doi:10.1016/j.jamda.2025.105831)
Supplement: Supp material [file NIHMS2110777-supplement-Supp_material.docx]

**Search Strategy**

Ovid MEDLINE(R) <1946 to April 10, 2024>

1 (smart glass* or smartglass* or smart eyeglass* or wearable computing device or augmented reality glass* or augmented reality goggles or head mounted display*).mp. [mp=title, book title, abstract, original title, name of substance word, subject heading word, floating sub-heading word, keyword heading word, organism supplementary concept word, protocol supplementary concept word, rare disease supplementary concept word, unique identifier, synonyms, population supplementary concept word, anatomy supplementary concept word] 1356

2 Smart Glasses/ 211

3 head worn display.mp. 13

4 wearable augmented reality.mp. 15

5 augmented reality display*.mp. 38

6 google glass*.mp. 146

7 1 or 2 or 3 or 4 or 5 or 6 1513

8 exp Dementia/ or dementia.mp. 250439

9 Alzheimer.mp. 133401

10 cognitive impair*.mp. 80549

11 cognitive dysfunction.mp. 52507

12 cognitive function*.mp. 73880

13 8 or 9 or 10 or 11 or 12 360607

14 7 and 13 26

15 cognition.mp. 239489

16 13 or 15 508468

17 7 and 16 71

18 17 not 14 45

19 (elder* or senior* or old* adult or old* people or aged or geriatric* or frail).mp. [mp=title, book title, abstract, original title, name of substance word, subject heading word, floating sub-heading word, keyword heading word, organism supplementary concept word, protocol supplementary concept word, rare disease supplementary concept word, unique identifier, synonyms, population supplementary concept word, anatomy supplementary concept word] 6027486

20 17 and 19 20

24 results exported on 6/13/24

Changed ‘cognition’ to ‘cognitive’- 12 new results exported on 7/11/24
